# A Review of the Mysterious Roles of the Autonomic Ganglia Considered as Deep Intelligence Agency in the United States of the Brain

**DOI:** 10.5152/eurasianjmed.2023.23284

**Published:** 2023-12-01

**Authors:** Mehmet Dumlu Aydin

**Affiliations:** Department of Neurosurgery, Atatürk University Faculty of Medicine, Erzurum, Türkiye

**Keywords:** Autonomic ganglia, deep intelligenct agency, deep state of brain

## Abstract

For centuries, the brain has been considered a single organ from an anatomical and functional perspective. However, while the cerebral cortex, consisting of many lobes and lobules, generally creates voluntary actions, autonomous parts of the brain also carry out vital activities such as survival, reproduction, and nutrition. The functions of the group of organs that carry out basic vital activities are modulated by autonomous ganglia that work like the deep intelligence networks of the brain. Information and energy packets produced as different molecules in vital organs and sent to the autonomous ganglia are decoded. These packages are then made available for use by cells, tissues, and organs. This deep information, purified and summarized by the autonomic ganglia, is presented to the cerebral cortex after passing through the control of the brainstem and insula. As a result, the entire brain and all the organs under its control decide together what to do. From this conceptual perspective, the brain is a united states group with different states under its management; the autonomic ganglia can also be thought of as the brain’s deep intelligence networks.

## Introduction

The brain has been thought of as a single organ from ancient times to the present. However, the brain is a whole consisting of many subunits that are connected to each other through very deep networks of anatomical and physiological relationships and work as semi-independent states within themselves. The cerebral cortex modulates conscious mental processes processed by light and sound signals. However, nutritional and reproductive functions, which are processed by smell and taste signals, are carried out by autonomous ganglia, which are the brains of each organ, such as the hypothalamus, pituitary, pineal, and brainstem nuclei, under the control of the brain’s unconsciously working insular cortex. Autonomic ganglia direct and manage the organs in the organism, which operate with subconscious and unconscious commands, with endless flow of information. Autonomic ganglia manage the organs in the organism, which they operate with subconscious and unconscious commands with endless flow of information. For this reason, they can be called deep intelligence networks of the brain. Autonomic ganglia decode the information flowing in the form of atoms or molecules from the organs they control by purifying it from its raw materials. Then, it encodes this information with irrational and complex numbers and presents it to the spinal cord, brainstem, and insular cortex. Here, the information reaching a general conclusion is sent to the cerebral cortex. The cerebral cortex informs the brain’s intelligence agency what to do based on this latest information. Autonomic ganglia resemble fault lines under the cerebral cortex. A small crack in the fault lines will cause software and hardware earthquakes in the brain. The cause of many diseases of unknown origin may be due to anatomical, histological, physiological, and biochemical disorders of the autonomic ganglia. In a way, they are the black boxes of the brain. The actions of eating, drinking, and breeding, which are necessary for life and reproduction, the process of protection against diseases, and the activities related to social life are all created by the information obtained from inner and outer space by the deep intelligence agency consisting of autonomous ganglia. For this reason, autonomous ganglia are the producers of countless abstract software such as mind, heart, conscience, love, and hate. For example, these deep intelligence networks of the brain are responsible for disrupted blood chemistry when the carotid bodies do not work, blood pressure changes when the pressure corpuscles do not work, metabolism when the smell and taste buds do not work, and relationships between spouses and even internationally. For a healthy personal, familial, social, and universal order, these skin intelligence networks consisting of autonomous ganglia must have excellent software and hardware.

### Research Consequences

In this study, brief information will be presented about the identities of the autonomic ganglia, which incomprehensibly manage almost an endless variety of biochemical, physiological, histological, anatomical, and mental activities without reflecting them to consciousness. The data is a summary and critique of our own published experimental work. The behaviors of the autonomous ganglia, or the deep intelligence agency of the brain, described here are examined, and their relationships within themselves, together and with the organs they manage, and between the brain and the brains in times of war and peace are examined. For example, the response of the testicles to the interruption of the olfactory signals that the Onuf’s nucleus loves so much is to produce defective sperm and thus consume the species. The interruption of taste signals results in diseases that progress with either cachexia or obesity. In summary, while autonomic ganglia are the defense lines of the brain, they can also turn into fault lines in the brain when they become involved in an interganglionary war.

### A Brief Analysis of Autonomic Ganglia in the Light of Our Experimental Perspectives

The summaries were made by examining our own experimental publications together with the literature. Those who want to access detailed information should look at the literature in the article.

### Carotid Bodies

**Figure 1.**
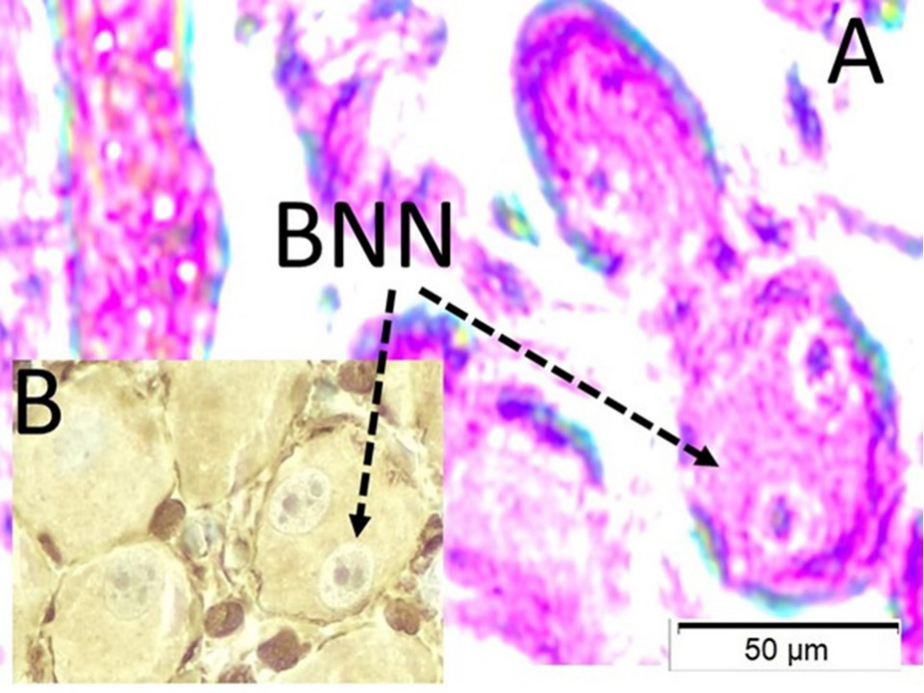
Histological appearances of binuclear neurons (BNN) of carotid body (LM, HE, ×20/A; GFAP, ×20/B).

Carotid bodies are the most important pH modulators by way of detecting the external carotid artery and internal carotid artery. The glossopharyngeal nerve and carotid body network are the main parts of the blood pH regulation.^1^ Ischemic insult carotid bodies could be converted to renormalization due to retrograde blood flow via vertebrobasilar territory to carotid territory with a polygon of Willis.^2^ The more neurodegenerative pathologies of glossopharyngeal nerves^3^ especially binuclear neuronal neurodegeneration in carotid bodies can lead to systemic acidosis.^4^ Neurodegeneration of carotid bodies can also cause decreased cerebrospinal fluid acidosis-related ependymal cell degeneration and dangerous insult in the brain following subarachnoid hemorrhage.^5^ Acidosis occurring as a result of carotid body damage during cervical trauma ^6^ or surgical interventions may lead to choroid plexus damage^7^. Carotid body injuries can cause respiratory disturbances and respiratory arrest during subarachnoid hemorrhage.^8^ It produces death charts by disrupting the chemistry of all body fluids, including the malfunction of the carotid body. Figure 1 shows the histological appearances of binuclear neurons of carotid bodies.

### Nodose Ganglia

Nodose ganglia are conveyed from visceral afferent signals via vagal nerves to the brainstem and higher brain centers such as the insula. The nodose ganglia, stellate ganglia, and intrinsic cardiac ganglia disturbances can lead to burned neurocardiac web syndrome,^9^ neurogenic stunned myocardium,^10^ acutely-developed nontreatable hyperglycemia,^11^ pulmonary lymph node infarts,^12^ coronary vasospasm^13^ and also cardiac arrest following subarachnoid hemorrhage.^14^ The low neuron density of the hilar parasympathetic ganglia of vagal nerves may be responsible for pulmonary artery vasospasm and neurogenic lung edema following subarachnoid hemorrhage.^15^ The neurodegeneration of the parasympathetic intrinsic cardiac ganglia may cause cardiac arrest and sudden death following subarachnoid hemorrhage.^9^ Vagal nerves and their thermoreceptors in the choroid plexus regulate brain temperature.^16^ Cardiac ganglia should be considered as rechargeable batteries of the heart. For that reason, intracardiac ganglia numbers and/or cardiac ganglia neuron density have important roles in cardiac survival following brain death after subarachnoid hemorrhage.^17^ The low neuron density of the intracardiac ganglia is a potential risk factor for atherosclerotic plaque development in cholesterol-fed rabbits.^18^ Disinformation of the deep intelligence agency of the nodose ganglion can cause dangerous cardiorespiratory disabilities during subarachnoid hemorrhage.

### Petrosal Ganglia

Baroreceptor reflexes are mainly modulated by the nerve terminals of the glossopharyngeal and vagal nerves located in the petrosal ganglions.^19^ Because the petrosal ganglion regulates blood pressure, petrosal ganglion ischemia or low neuron density of petrosal ganglia can lead to hypertension during subarachnoid hemorrhage.^20^ Aydin et al^21^ showed that the essential mechanism of rapidly decreased blood pressure with the application of sublingual nifedipine may also result from the direct stimulation of taste buds innervated by the glossopharyngeal nerves. Insufficient information on the petrosal ganglia can cause dangerous hypertension following subarachnoid hemorrhage.

### Pterygopalatine Ganglions

A pterygopalatine ganglion is also a parasympathetic ganglion, and secretory fibers travel with the deep branches of the trigeminal nerves. The pterygopalatine ganglion innervates the mucous membranes of the eyes, ears, nose, mouth, tongue, tonsils, soft palate, uvula, upper lip, gums, and upper part of the pharynx.^22^ Pterygopalatine ganglion lesions cause dry mouth, dry nose, and dry eyes. 

### Ciliary Ganglions

The ciliary ganglion is a sensor that adjusts the digital and optical zoom of the eye and is almost a parasympathetic power bank of the oculomotor nerves. In case of deficiency, visual disturbances and photophobia occur. Meningitis,^23^ subarachnoid hemorrhage,^24^ and aneurysms of posterior communicating arteries^25^ can cause cellular injury and necrosis on both oculomotor nerves and ciliary ganglions. Oculomotor nerve and ciliary ganglion circuitry insults should be considered as an essential factor for photophobia due to increased photon transport via dilated pupils.^26^ Ciliary ganglion dysfunction disrupts the digital and optical zoom settings of the eyes and destroys the brain’s Photoshop system.

### Trigeminal Ganglions

**Figure 2.**
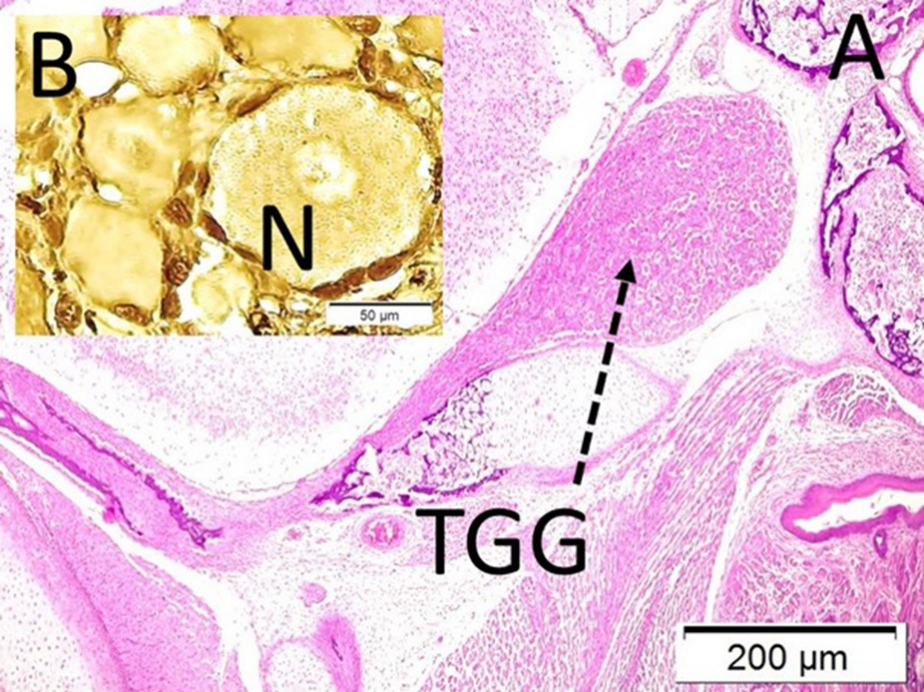
Histological appearances of the sphenoid base, trigeminal ganglion (TGG), and neurons of TGG (B) are seen in rat pub (LM, HE, ×4/A; GFAP - ×20/B).

The trigeminal system regulates intracranial, intraocular, and inner ear pressure and temperature. For trigeminal network, injuries lead to increased intraocular pressure,^27^ scalp ischemia and hair loss,^28^ hypoacusia,^29^ decreased cerebral blood flow due to anterior choroidal artery vasospasm following subarachnoid hemorrhage.^30^ Trigeminal ganglion dysfunctions cause increased intracranial temperature, increased intracranial–intraocular pressure, and acid–base balance disturbances. Figure 2 shows the histological appearances of the trigeminal ganglion on the sphenoid bone.

### Onuf’s Nucleus

**Figure 3.**
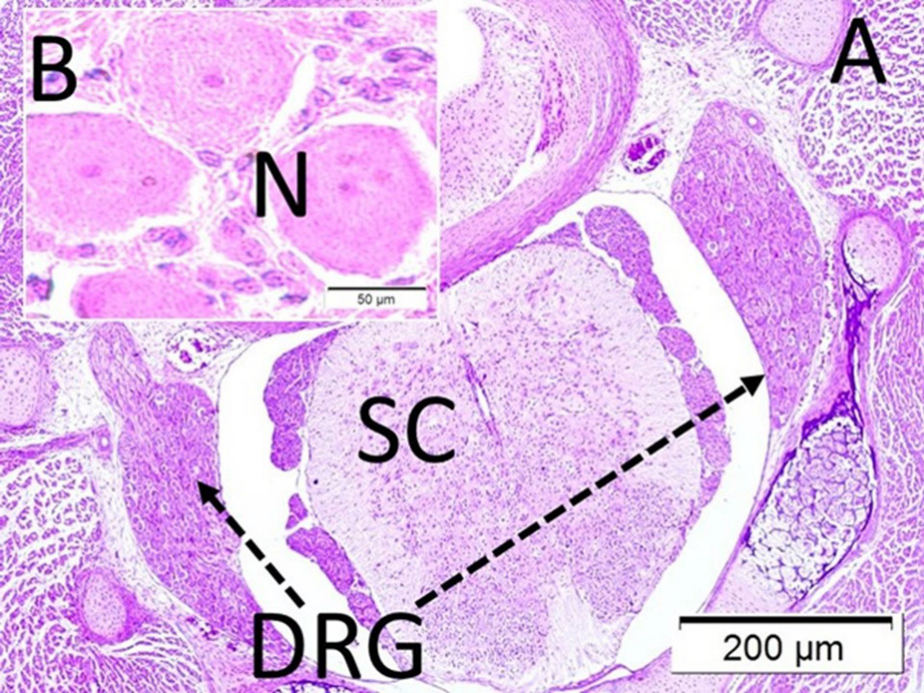
Histological appearances of the spinal cord (SC), dorsal root ganglions (DRG), and neurons of DRG (B) are seen in rat pub (LM, HE, ×4/A; ×20/B).

The Onuf’s nucleus is a parasympathetic nucleus and is located in the medullary conus. Onuf’s nucleus sends its signals to the distal colon and genital organs via the pudendal, pelvic, and hypogastric nerves in both men and women. In females, these organs are also innervated by the vagal nerve. Lumbosacral pathologies can lead to infertility only in men, not women. Onuf’s nucleus lesions may be responsible for decreased sperm numbers^31^ and decreased orgasmic pleasure due to urethral taste buds denervation.^32^ Because there is an important bridge between olfactory nerves and Onuf’s nucleus,^33^ olfactory bulb lesions may cause intestinal and genital organ disabilities. Onuf’s nucleus pathologies can lead to Auerbach/Meissner ganglia degeneration like as Hirschsprung,^34^ mesenteric artery vasospasm^35^ following subarachnoid hemorrhage. The following literature should not be forgotten. Adamkiewicz artery supplies the Onuf’s nucleus, pudendal, pelvic, and hypogastric nerves, which are branches of the sacral parasympathetic plexus. Onuf’s nucleus–pudendal nerve ganglia complex degeneration secondary to vasospasm of the Adamkiewicz artery may be a cause of urinary retention,^36^ disabled sexual relations, and reproduction following Onuf’s nucleus insults. Figure 3 shows the Auerbach ganglia innervated by Onuf’s nucleus in rats.

### Adamkiewicz Artery-Dorsal Root Ganglions

**Figure 4.**
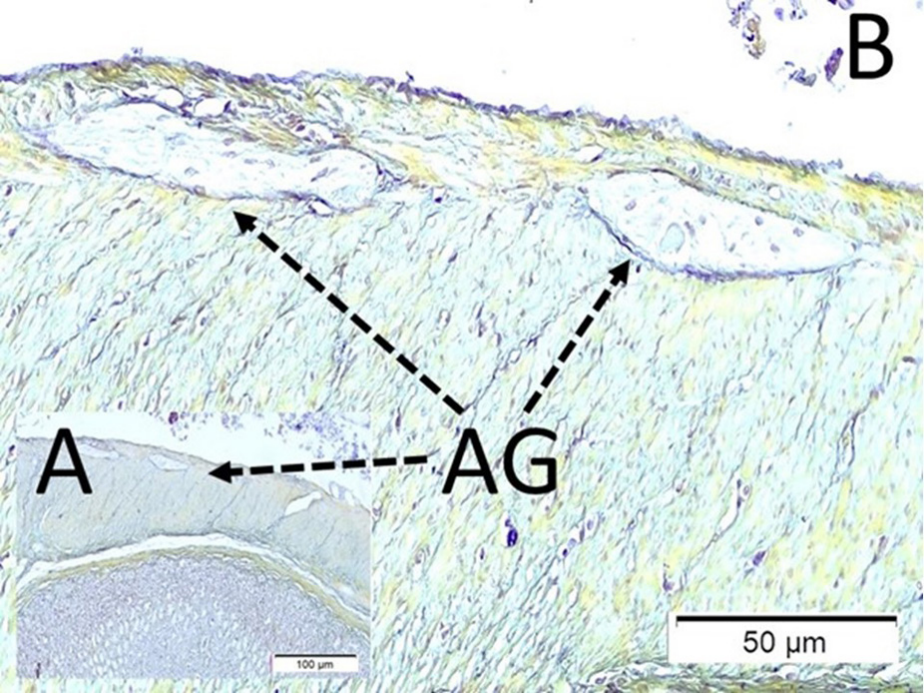
Histological appearances of bowel and Auerbach ganglia (AG) neurons of bowels are seen in rat pub (LM, NSE, ×10/A; GFAP - ×20/B).

Increased neurodegeneration of the lumbar dorsal root ganglion may be responsible for femoral artery vasospasm,^37^ Hirschsprung-like diseases,^34^ and second motor neuron diseases.^38^ The cervical dorsal rootganglia degeneration may lead to basilar artery vasospasm after subarachnoid hemorrhage.^39^ The denervation injuries of genital taste roses due to pudendal nerve ganglia insults may be responsible for orgasmic sensation disabilities.^40^ The decreased blood supply of the lower spinal cord, which is maintained by the Adamkiewicz artery, may lead to spinal cord ischemia during subarachnoid hemorrhage.^41^ Cervical spinal nerves and their ganglia insults cause cardiorespiratory disturbances and sudden death during subarachnoid hemorrhage.^42^ External carotid artery vasospasm-related ischemic changes occur in the carotid body and sympathetic ganglia.^43^ Adamkiewicz artery spasm-induced lumbosacral spinal cord pathologies can cause bowel dilatation.^44^ Phrenic nerve root ischemia may be an important causative factor in respiratory deterioration following subarachnoid hemorrhage.^45^ The insults of sensory fibers of upper cervical nerves cause severe vasospasm of anterior spinal arteries during subarachnoid hemorrhage.^46^ The interruption of the neural network between arterial nervorums and nervi arteriorums in the sciatic nerves may be responsible for sciatic nerve injury.^47^ Somatosensitive cortex lesions may cause descendent dorsal root ganglion degeneration-induced spasticity and/or ileus.^48^ Monopolar electrocauterization can lead to neurodegeneration in the spinal ganglia.^49^ The dorsal root and cranial nerve ganglia are the intermediate parliaments between the brain and deep intelligence networks. Figure 4 shows the histological appearances of dorsal root ganglions.

### Sympathetic Ganglions

Sympathetic ganglia are the main military element of the intelligence agency. The internal war of this system destroy the organism. The discordances among vagal network/stellate ganglion and intrinsic cardiac ganglia following subarachnoid hemorrhage cause neurodegeneration of the cardiac ganglia and sudden death.^9^ Anteroposterior cerebral blood flow is regulated by the basilar arteries. 

### Thoracic Sympathetic Center and Stellate/Superior Cervical Sympathetic Ganglia

Superior cervical sympathetic ganglia fibers have a vasoconstrictor effect on the basilar arteries. There is an inverse interaction between the degenerated neuronal density in the superior cervical sympathetic ganglia and the degree of basilar arteries vasospasm. Ischemic neurodegeneration of the superior cervical sympathetic ganglia may protect the basilar arteries from spasm after subarachnoid hemorrhage.^50^ Fatal pulmonary edema and intrapulmonary hemorrhages are significant complications of stenoocclusive carotid artery disease.^51^ The neurodegeneration of the thoracic sympathetic nuclei may lead to low-frequency heart rhythm abnormalities,^52^ partially involved in the development of neurogenic stunned myocardium or takotsubo cardiomyopathy^10^ and permanent miosis following subarachnoid hemorrhage.^53^

The superior cervical ganglia ischemia due to carotid stenosis may cause dangerous vasodilatation of basilar arteries.^54^ A high density of degenerated neurons in the stellate ganglia may provoke excessive sympathetic hypoactivity-related cardiac damage and bradyarrhythmias due to carotid artery diseases.^55^ High neuron density in the stellate ganglia may prevent basilar artery dilatation and aneurysm formation in the posterior circulatory arteries in stenoocclusive carotid artery diseases.^56^ High degenerated neuron density of the ciliary ganglion or high neuron density of the superior cervical sympathetic ganglia should be considered an important factor for permanent pupil dilatation.^57^ High neuron density of stellate ganglion may cause neurogenic pulmonary edema development during subarachnoid hemorrhage.^58^ The high neuron density of stellate ganglion may play an important role in the development of basilar artery vasospasm during subarachnoid hemorrhage. The beneficial effect of sympathectomy for the prevention of cerebral vasospasm may be explained through this mechanism.^59^ This intelligence agency of the brain can collect information from the deepest networks as well as leak its information. With these effects on the face and eyes in cases of anger or joy, sympathetic ganglia can reveal deep secrets in terms of diplomacy by dangerously displaying information about the brain exchange system, senate, and deep management. 

### Celiac Ganglia

The celiac ganglia are responsible for the sympathetic innervation of abdominopelvic organs. The celiac ganglia are located in the inferior level of the celiac artery. The celiac ganglia send their postganglionic fibers as a perivascular plexus around mainly mesenteric arteries. The celiac ganglia modulate the innervation of the distal esophagus, stomach, duodenum, small intestines, proximal colon, adrenal glands, pancreas, spleen, liver, and biliary system. Various abdominopelvic organ disorders, nutritional and reproductive disorders, sphincter insufficiencies, and ejaculation disorders frequently occur in celiac ganglion deficiencies.^60^ The celiac ganglia are the basic intelligence gladiators of abdominal organs.

### Taste Buds-Geniculate Ganglia

**Figure 5.**
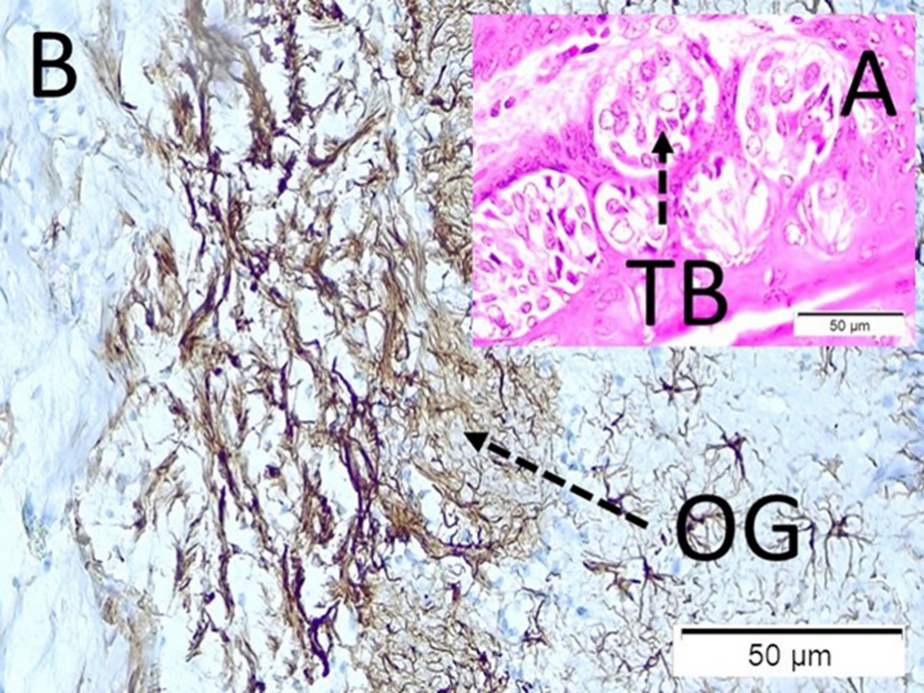
Histological appearances of tongue taste buds (TB) and olfactory nerve tufts-glomerulus (OG) are seen in rat pub (LM, HE, ×20/A; GFAP - ×20/B).

Newly detected intrapancreatic taste buds like cell clusters have an important role in the regulation of blood glucose levels in the pancreas.^61^ Probably, taste buds of the tongue stimulate geniculate ganglion neurons to inform insulin-secreting branches of vagal nerves to secrete insulin from Langerhans Islands.^62^ Congenital deficiency in the number of taste buds may be responsible for diabetes. If they are given the wrong signals with artificial foods, they take heavy revenge on those who cheat by producing severe metabolic diseases. Figure 5A shows the taste buds of a rat. 

### Olfactory Tufts-Basal Ganglia

Recently, important bridgings have been discovered between olfactory nerves and various neural webs,^33^ Olfactory bulbectomy may lead to the degeneration of substantia nigra,^63^ Raphe nuclei degeneration,^64^ denervation injury of Peyer’s patches,^65^ mammary gland atrophy,^66^ hypothyroidism,^67^ neuronal degeneration in habenular nuclei,^68^ Onuf’s nucleus degeneration induced reduced sperm numbers.^69^ While these ways protect the organism from invisible troubles, they can also be spies that host coronavirus disease 2019 (COVID-19)-type viruses. Figure 5B shows the olfactory glomerulus of a rat. 

### The Importance of Autonomic Ganglia in Epidemic Diseases: Coronavirus Disease 2019 Pandemic and Future Pandemics

Smell and taste signal systems are the most effective radars used against unconscious biological, chemical, and physical dangers. Unfortunately, the olfactory and taste nerves that manage smell and taste signals have been seriously damaged by the disinfectants used during the COVID process. Evaporating alcohol from disinfectants reaches the subarachnoid space through fila olfactoria and damages the choroid plexuses, which play the role of the brain’s spleen, liver, lung, kidney, etc., which may pave the way for serious dangers such as Alzheimer’s and Parkinson’s disease.^70^ Again, due to the gonadotropic and olfactotropic properties of COVID-19, localization of the olfactory and taste nerves will most likely cause important complications such as atrophy of the endo-exocrine glands,^67^ depression,^68^ atrophy of the mammary glands^66^ intestinal immune deficiency^65^ dysorgasmia,^32^ and spermatogenesis disorders.^69^ I think the complications will not be limited to this.

## Conclusion

Currently, advanced technology has been used in medical practice;^71^ however, despite the increased use of technology, the proper function of the autonomic ganglia is not well-known.

Each organ has its own function, and the autonomous ganglia located in the organ hilus modulate the events of life, reproduction, health, and death within that organ itself in the relations between organs and their relations with the external universe, thanks to the biochemical, microbiological, histological, and anatomical staff of that organ. This structure, which we call the deep intelligence agency of the brain, maintains peace within itself or among existing ones, usually through operations; sometimes they may fight by choosing military means. In this second case, a computer’s autonomous ganglia, data, and software are damaged. It shows symptoms of neurodegenerative, hormonal, immunological, and cancerous diseases that are very difficult to treat. Therefore, we say that peace in the deep intelligence networks of the brain is the guarantee of peace in the body, peace in the human world, and even peace between all living things.

## Future Insights

War and peace between autonomic ganglia or between these ganglia and the organs they are connected to is caused by incomplete or faulty decoding of the signals coming from these ganglia. Artificial foods of artificial life disrupt first the hardware and then the software in these ganglia, causing false information to spread in the deep intelligence networks of the brain. Thus, the organism falls into severe chronic destruction or death.
